# Dual-Band, Dual-Mode, Circularly Polarized Fully Woven Textile Antenna for Simultaneous Wireless Information and Power Transfer in Wearable Applications

**DOI:** 10.3390/s26010030

**Published:** 2025-12-19

**Authors:** Miguel Fernández, Carlos Vázquez, Samuel Ver Hoeye

**Affiliations:** Electrical Engineering Department, Oviedo University, E33203 Gijón, Spain; vazquezcarlos@uniovi.es (C.V.); versamuel@uniovi.es (S.V.H.)

**Keywords:** antennas, rectenna, rectifier, textile technology, wearable, wireless power transfer

## Abstract

In this work, a dual-band, dual-mode, circularly polarized fully woven textile antenna with capability for Simultaneous Wireless Information and Power Transfer (SWIPT) in wearable applications is presented. The power and the data transfer modes work at 2.4 and 5.4 GHz, respectively. The radiating element is based on a square patch with an asymmetrical U-shaped slot and a chamfered corner. A single-diode rectifier, required for the power transfer mode, is mounted on a carrier thread and then connected to the antenna through a T-match network located at one of the patch corners. This feeding technique simultaneously provides complex conjugate matching to the rectifier and circular polarization. On the other hand, a coaxial probe port is used for the data transfer mode. A prototype was implemented and experimentally characterized. Regarding the power transfer mode, the measured RF-DC conversion efficiency is about 50% when the available power at the rectifier input is −10 dBm, and the axial ratio is smaller than 3 dB. In the data transfer mode, the antenna gain and the axial ratio are 0 and 2 dB, respectively. The experimental results are in good agreement with simulations, validating the proposed structure and design methods, and they are comparable to the state of the art for textile antennas/rectennas. Furthermore, the combination of the fully woven technology and the proposed single-layer layout provides a large degree of integration and robustness, which are valuable characteristics for wearable devices.

## 1. Introduction

In recent years, wearable technologies have attracted the interest of the scientific and industrial communities because of the large number of potential applications related to the remote monitoring of a variety of vital signs and in the fields of sports [1] and healthcare [2,3], among many others. However, there are still some challenges to be addressed in the design of true wearable devices capable of providing a comfortable user experience related to the integration and the DC powering [4]. In this context, the Simultaneous Wireless Information and Power Transfer (SWIPT) approach is considered to be promising, since it integrates both the power transfer and communication capabilities in the same radiating structure, increasing the compactness of the final device [5,6].

Regarding DC powering, the use of batteries in wearable devices negatively impacts the user experience, since they are difficult to integrate, increase the size and weight and impose charging or even replacement cycles. To address this challenge, the Wireless Power Transfer (WPT) approach has been proposed as a viable power supply technology, which is especially suited for low-power devices. It is based on rectifying an incoming RF signal from a dedicated narrowband RF transmitter, then using the resulting DC voltage to power the sensing and communications circuitry. Since the parameters of the dedicated RF transmitter are stable and known to the designer, the performance of the system mainly depends on the rectenna behavior, and more specifically, on the impedance matching between the rectifier and the antenna [7,8].

Considering the integration challenge, most of the effort should be focused on the design of the radiating structure, since it is usually the largest part of the device. The use of textile substrates provides some valuable characteristics for the design of wearable devices, such as flexibility, breathability, and ease of integration in fabric. A number of different nonwoven approaches have been described in recent years, including the simple concealment of a flexible antenna inside a fabric pocket [9], stacking several shaped conductive and dielectric fabric pieces [10,11,12], hybrid implementations combining textile and conventional RF substrates [13,14], and embroidering with conductive thread on dielectric fabric sheets [15,16,17]. These works report acceptable electrical performance, but the achieved robustness and degree of integration are limited because, in general, the presented technologies involve the use of different independent layers attached between them, and the integration of the circuitry is made by soldering lumped electronic components or rigid PCB parts to the textile structure.

The use of textile fully woven technology has also been reported [18]. When compared to nonwoven devices, it exhibits improved robustness, ease of integration with larger fabric pieces, and the capability of large-scale production using processes and machinery from the textile industry. This promising technology was successfully applied, in combination with electronic circuitry, for the development of UHF RFID tags [19] and rectennas [20,21,22]. In these cases, the required circuitry was previously mounted on carrier threads and then woven with the rest of the textile structure, achieving improved robustness and the largest degree of integration described to date while preserving the advantages of fully woven technology.

Different strategies for the design and implementation of the SWIPT approach have been presented. On the one hand, the use of single-band, dual-port antennas [21,23,24,25,26] has been considered. In these cases, the rectifier and the communication circuitry are directly connected to two different ports of the antenna, or through two isolated ports of a hybrid coupler, achieving linear polarization when the two working modes work simultaneously, or circular polarization when they are multiplexed in time. On the other hand, multi-band design techniques [27] have also been exploited. Dual-band, dual-port SWIPT antennas [12,28,29,30], in which the power and the data transfer are performed at different frequencies, were also presented, improving the isolation between the two working modes. With the exception of [12,21,26], all the previously cited SWIPT devices were implemented in conventional rigid RF substrates and, in general, they present complex multilayer structures and require the use of large impedance matching networks to connect the rectifier, which makes them unsuitable for wearable applications.

In this work, a textile fully woven dual-band, dual-port SWIPT antenna is presented. The power and the data transfer modes operate at 2.4 and 5.4 GHz, respectively, both with circular polarization, in order to minimize the negative impact of potential polarization mismatch between the transmitter and the receiver antennas. The radiating structure is based on a square patch with a U-shaped slot. For the power transfer mode, a single-diode rectifier is connected to the antenna through a T-match network located at a patch corner, while a conventional coaxial probe is used for the data signal. The resulting layout is compact and geometrically simple, which makes it compatible with fully woven technology. The measured RF-DC conversion efficiency at the power transfer mode is aligned with the state of the art for textile rectennas, while the radiation properties at the two working modes are adequate for wearable applications.

## 2. Materials and Methods

### 2.1. SWIPT Antenna Description

**Figure 1:**
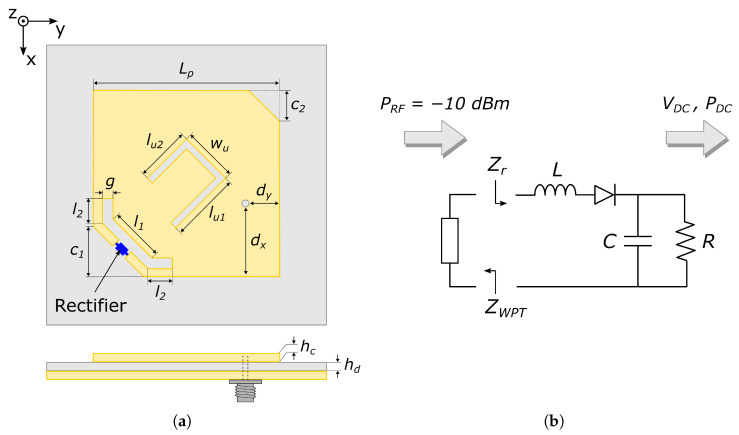
(**a**) Layout of the proposed SWIPT antenna. (**b**) Single-diode rectifier circuit diagram.

The structure of the proposed SWIPT antenna, which is composed of a dual-band, dual-port radiating structure and a single-diode rectifier, is shown in Figure 1a. The radiating element is based on a nearly square patch with an asymmetric U-shaped slot and a chamfered corner fed through two independent ports. For the WPT mode, the rectifier is connected to the antenna through a T-match network [31] located at one patch corner, which simultaneously provides complex conjugate matching to the rectifier and circular polarization. Regarding the data transfer mode, a 50 Ω coaxial probe is added to connect the information signal. The described structure is low profile and the antenna layout is compact and simple enough to be compatible with the fully woven technology. Furthermore, the inherent ground plane isolates the antenna from the underlying objects, limiting their impact on the system performance, which is a very valuable characteristic when considering wearable devices.

Figure 1b schematizes the rectifier topology. It is composed of a single BAT15-04 Schottky diode, from Infineon Technologies, followed by a shunt capacitor, C=1 nF, to short circuit the RF signal at the diode output. The input series inductor, L=6.8 nH, is introduced to partially compensate for the large capacitive part of the rectifier input impedance, $Z_{r}$, in order to simplify the impedance matching to the antenna. The output resistor, R=5.1kΩ, is used to emulate the rectifier load. Its value was selected based on the analysis presented in [20] as a trade-off between the output DC voltage, $V_{DC}$, and the RF-DC conversion efficiency, $\eta_{RF-DC}$. The rectifier is first mounted on a carrier thread which is then woven with the rest of the textile structure, obtaining a large degree of integration and robustness, since no soldered connections to textile pads are required.

### 2.2. Fully Woven Structure and EM Model

**Figure 2:**
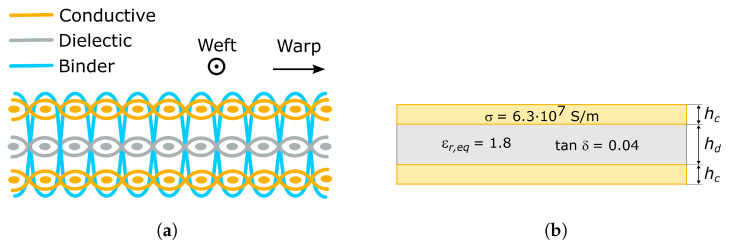
(**a**) Fully woven textile structure of the substrate. (**b**) Homogeneous-layer equivalent EM model.

The three-layer woven structure used as the basis for the antenna development is schematized in Figure 2a. It includes two conductive layers separated by a dielectric layer. As in a conventional rigid RF substrate, the antenna layout is patterned on the top conductive layer while the other acts as the ground plane.

The woven structure is conceived to be manufactured in a single step in an industrial loom. Thus, it is composed of warp and weft threads, which are perpendicular to each other. The dielectric layer is woven with polyester threads composed of two twisted yarns. The conductive layers are woven with ELITEX 117/f17 2ply threads, from Imbut Gmbh (Greiz, Germany), formed by two twisted yarns, each one with 17 filaments extruded from polyamide. The threads are coated with a 1 μm thick silver foil, which provides a 70 Ω/m linear resistance. The dielectric and conductive layers are held together using dielectric binder threads, identical to those used in the dielectric layer. The binder threads are added during the weaving process, resulting in an indivisible fabric piece. This approach enables large-scale production and provides improved robustness when compared to other nonwoven alternatives, in which several independent fabric pieces have to be attached together.

The direct EM modeling of the fully woven structure schematized in Figure 2a is a complex task, because of the geometrical complexity, and the complexity is further increased due to the fact that the represented threads are composed of several filaments. Therefore, from a computational point of view, the mesh density required to accurately solve the problem represents a very high computational burden. Several strategies have been developed to overcome this issue while preserving accuracy [18]. These rely on replacing each of the three fabric layers with a homogeneous material with equivalent EM properties, as schematized in Figure 2b.

The dielectric woven layer is modeled as a homogeneous sheet with the same thickness, $h_{d}$. The equivalent dielectric permittivity, $\varepsilon_{r,eq}$, and loss tangent, tanδ, were estimated from the experimental characterization of a transmission line section and a resonator implemented in the same textile structure. The obtained equivalent dielectric permittivity is $\varepsilon_{r,eq}\approx 1.8$, smaller than the polyester value because of the air gaps contained in the woven structure. On the other hand, the obtained equivalent loss tangent value is tanδ≈0.04.

In the case of the conductive layers, electric currents can flow in any direction due to the large number of contacts between warp and weft threads generated at the weaving stage. In addition, the skin depth on the silver coating at the working frequencies is slightly larger than the 1 μm coating thickness. Therefore, the conductive woven layers can be modeled as a homogeneous conductive sheet with the same thickness as the silver coating, $h_{c}=1\;\mu m$, and the same conductivity as silver, $\sigma =6.3\times 10^{7}$ S/m.

### 2.3. Rectifier Analysis

The rectifier schematized in Figure 1b was analyzed through nonlinear simulations based on the Harmonic Balance (HB) technique implemented in the commercial CAD software package Advanced Design System v2015.01, from Keysight Technologies. The rectifier input signal is supposed to be generated by a dedicated WPT signal source, with frequency $f_{RF}=2.4$ GHz. The available power at the rectifier input is considered to be $P_{RF}=-10$ dBm, which is a realistic value when considering the EIRP limitations for indoor transmitters and the free-space propagation losses.

Under the indicated working conditions, the calculated input impedance is $Z_{r}=16-j70\;\Omega$, the output DC voltage and power are $V_{DC}=0.55$ V and $P_{DC}=60\;\mu$W, respectively, which provides a RF-DC conversion efficiency $\eta_{RF-DC}=60\;\%$:

(1) $$\eta_{RF-DC}\left(\%\right)=\frac{V_{DC}^{2}}{R\times P_{RF}}\times 100$$

**Figure 3:**
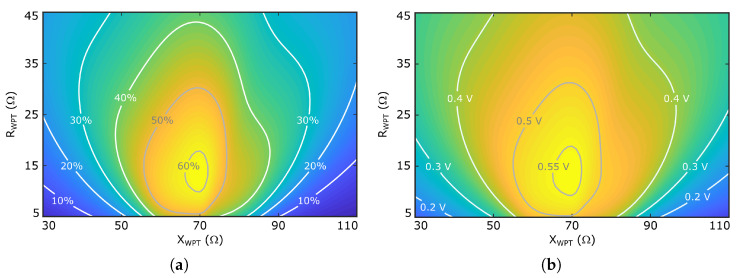
Effect of the impedance mismatching between the rectifier and the WPT port of the antenna on the whole system performance. (**a**) RF-DC conversion efficiency. (**b**) Rectified voltage $V_{DC}$.

In order to obtain a whole antenna-rectifier system efficiency close to that of the rectifier, complex conjugate impedance matching between the rectifier and the antenna WPT port should be achieved, $Z_{WPT}=Z_{r}^{*}=16+j70\;\Omega$. Fully woven technology imposes fabrication constraints which must be considered during the design process. Since the geometry is composed of woven threads, dimensions can only be modified in discrete steps, associated with adding or removing individual threads. Thus, the geometric resolution is limited by the thread separation in the woven structure, which may, in turn, restrict the accuracy with which the desired input impedance, $Z_{WPT}$, can be realized. In addition, due to the flexible nature of the textile implementation, the structures may be subject to deformations under real use conditions, which may also affect the antenna input impedance. To estimate the impact of such effects, Figure 3 represents the evolution of $\eta_{RF-DC}$ and $V_{DC}$, when considering variations on the real, $R_{WPT}$, and the imaginary, $X_{WPT}$, parts of $Z_{WPT}$ about the optimum value. As expected, the maximum value of $\eta_{RF-DC}\approx 60\%$ is achieved when $Z_{WPT}=Z_{r}^{*}=16+j70\;\Omega$, and it reduces when either the real and/or the imaginary parts of $Z_{WPT}$ move away from that point. Nevertheless, note that the system tolerates variations of about 15 Ω in both impedance components, keeping the efficiency above 50%.

### 2.4. Antenna Design

The antenna is based on a square patch because of two fundamental reasons. On the one hand, this structure exhibits low profile and its layout requires only one patterned conductive layer, which makes it compatible with the fully woven textile technology. On the other hand, the inherent ground plane isolates the antenna performance from the underlying objects, which is a very valuable characteristic in wearable applications.

The patch is sized to resonate at 2.4 GHz at the fundamental mode, and it is excited through two different ports, each one associated with one of the two working modes, which present different electrical requirements. An asymmetric U-shaped slot and a corner chamfering were added to improve the antenna impedance matching and the radiation pattern characteristics.

#### 2.4.1. WPT Mode

The first design step is to optimize the antenna for the WPT mode, achieving the required input impedance value, $Z_{WPT}$, at the WPT port, evaluated at 2.4 GHz, to match that of the rectifier, $Z_{r}$. To do that, the port to which the rectifier will be connected is implemented as a distributed T-match network. This feeding technique provides an input impedance with low real part and large inductive imaginary part, required to match the input impedance of the rectifier. Furthermore, because of its particular location at one patch corner, two orthogonal modes with slightly different resonant frequencies, due to the length difference between the two patch diagonals, are excited, resulting in RHCP circular polarization [22]. An additional advantage of the proposed feeding structure is that the two port terminals are on the same plane, which simplifies the subsequent integration of the rectifier using a carrier thread.

**Figure 4:**
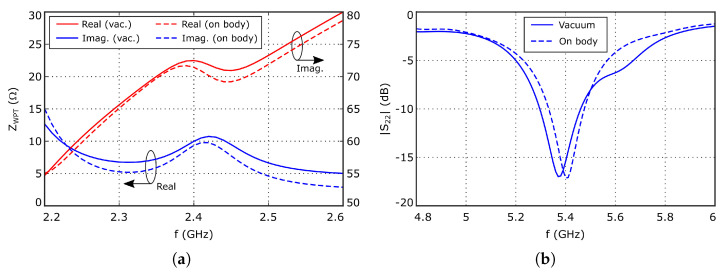
(**a**) Evolution of the input impedance of the antenna, $Z_{WPT}$, at the WPT port. (**b**) Reflection coefficient evaluated at the data 50Ω coaxial port.

A parametric analysis was conducted to study the influence of the geometrical parameters of the feeding structure on the antenna input impedance $Z_{WPT}$, while keeping constant the width of the arms, w=2 mm and the port gap p=6 mm, in order to generate a space large enough to place the rectifier. As reported in [20], the imaginary part of $Z_{WPT}$ increases with the total loop area, i.e., when increasing $l=l_{1}+2l_{2}$ and/or *g*. On the other hand, the real part of $Z_{WPT}$ increases with *g*, but it is weakly dependent on *l*, which provides a way to control both the real and the imaginary parts of the antenna input impedance. Figure 4a represents the evolution of the antenna input impedance with the frequency, showing a $Z_{WPT}\approx 10+j72\;\Omega$ at 2.4 GHz which, according to the data represented in Figure 3a, should provide a system RF-DC conversion efficiency larger than 55%. Furthermore, since the observed evolution with frequency is relatively smooth, both for the real and the imaginary parts, the impact of slight frequency deviations will be minimal. The optimized parameter values are summarized in Table 1.

**Figure 5:**
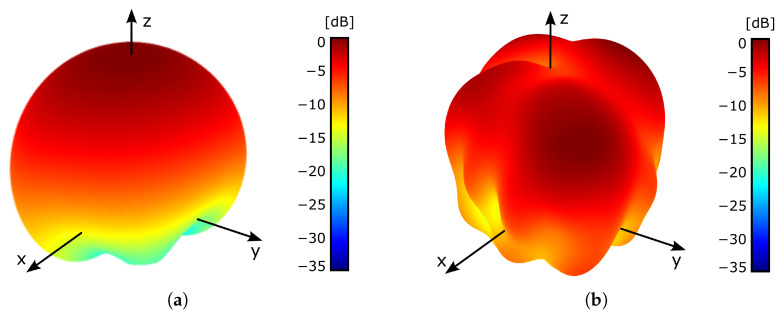
3D normalized radiation pattern. The radiating surface is located at the XY plane, centered with respect to the origin of the coordinate system. For the sake of representation clarity, values smaller than −35 dB have been clipped to −35 dB. (**a**) At 2.4 GHz, when exciting the WPT port. (**b**) At 5.4 GHz, when exciting the data transfer port.

**Figure 6:**
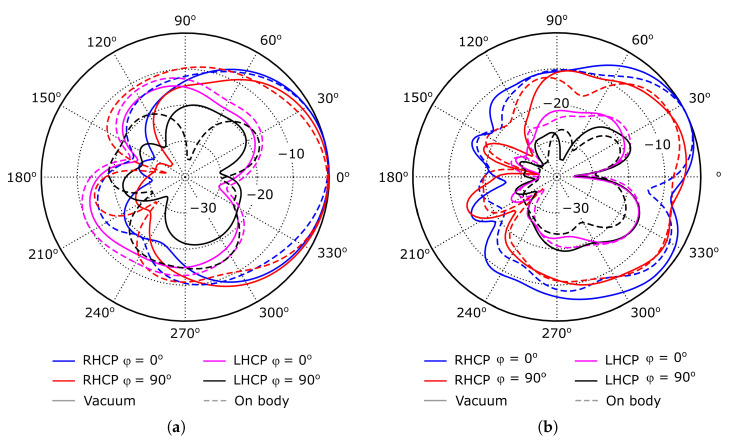
Normalized RHCP and LHCP components of the radiation pattern. For the sake of representation clarity, values smaller than −35 dB have been clipped to −35 dB. (**a**) At 2.4 GHz, when exciting the WPT port. (**b**) At 5.4 GHz, when exciting the data transfer port.

Figure 5a shows the simulated 3D radiation pattern, obtained when exciting the WPT port at 2.4 GHz. The radiating surface is located at the XY plane, centered with respect to the origin of the coordinate system. On the other hand, the simulated normalized RHCP and LHCP components of the antenna radiation pattern are represented in Figure 6a, showing the expected angular variation for a patch antenna. The maximum gain and radiation efficiency are about 0 dB and 30%, respectively, which are relatively small values for a patch antenna, mainly due to the large value of the substrate tanδ, and to its reduced thickness.

**Figure 7:**
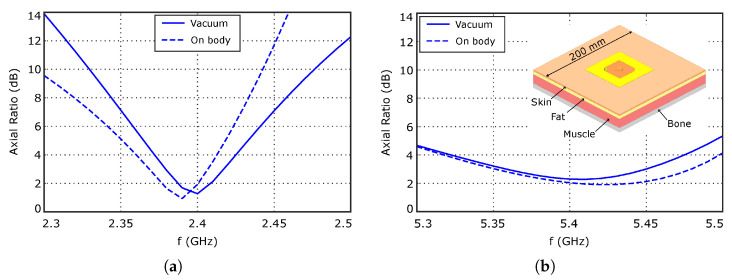
Evolution of the axial ratio, evaluated at $\theta =0^{o}$, with frequency. (**a**) WPT mode. (**b**) Data transfer mode. Inset: Antenna placed over the human body model.

**Figure 8:**
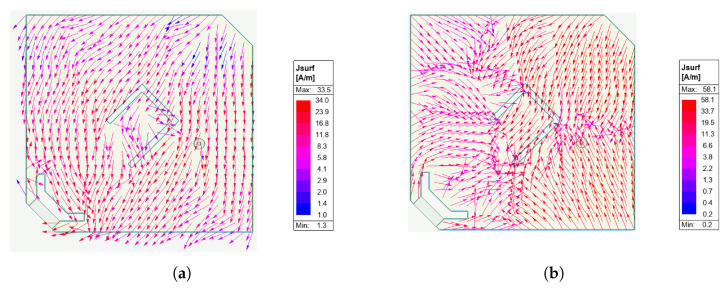
Simulated current distribution over the patch surface. (**a**) Evaluated at 2.4 GHz when exciting the WPT port. (**b**) Evaluated at 5.4 GHz, when exciting the data transfer port.

Figure 7a shows the evolution with frequency of the axial ratio, evaluated at $\theta =0^{o}$ direction. The value is smaller than 3 dB in the 2.37–2.42 GHz frequency range, in which the antenna input impedance varies from 8+j71Ω to 11+j72Ω. Considering the data represented in Figure 3a, the RF-DC conversion efficiency of the system should be larger than 50% in the considered frequency range. Furthermore, the reduced value of the axial ratio bandwidth at the WPT working mode will have low impact on the system performance, since the input signal at this working mode is an unmodulated carrier. Finally, Figure 8 shows the simulated current distribution on the patch surface at the two working modes, which confirms in a qualitative way the achieved polarization characteristics.

#### 2.4.2. Data Transfer Mode

Once the antenna has been optimized for the WPT mode, a 50Ω coaxial probe port to connect the data signal is added. The connection point of the port and the geometrical parameters of the slot were optimized to obtain good impedance matching and right-hand circular polarization at 5.4 GHz, while preserving the behavior of the WPT mode. Furthermore, the opposite corner to that at which the rectifier is connected was chamfered to improve the axial ratio.

Figure 4b represents the simulated reflection coefficient, $S_{22}$, evaluated at the data transfer port, showing acceptable performance. The simulated 3D radiation pattern, obtained when exciting the data transfer port at 5.4 GHz, is shown in Figure 5b. The radiating surface is located at the XY plane, centered with respect to the origin of the coordinate system. On the other hand, Figure 6b represents the normalized RHCP and LHCP components. In this case, the angular variation in the radiation pattern slightly differs from the expected shape for a patch antenna, due to a gain reduction at $\theta =0^{o}$. Nevertheless, since the proposed antenna is targeted for short-range applications, such gain reduction should have limited impact on the system performance. The maximum gain and radiation efficiency are about −1.5 dB and 24%, respectively, which are relatively small values for a patch antenna because of the same previously indicated reasons.

The axial ratio is shown in Figure 7b. It is smaller than 3 dB in the 5.35–5.45 GHz range, in which the reflection coefficient is smaller than −10 dB, ensuring a relatively large bandwidth, about 100 MHz, in terms of both impedance matching and axial ratio, which should be large enough for most of the targeted applications.

Finally, note that the presented design has been obtained as a trade-off between a large number of performance parameters for both working modes, which has been achieved using one single patterned conductive layer, in order to make it compatible with fully woven technology.

#### 2.4.3. Effect of the Human Body Proximity

The presence of a ground plane in the antenna structure is expected to minimize the influence of underlying objects on the antenna performance. Nevertheless, since the developed textile fully woven SWIPT antenna is intended to be used in wearable applications, the effect of the human body proximity was evaluated in simulation. The antenna was placed over a multilayer human tissue model composed of skin, fat, muscle, and bone layers, as schematized in the inset of Figure 7b. The thickness and electrical parameters of these layers are summarized in Table 2. The side length was set to 200 mm, which is slightly larger than 2λ at the lowest working frequency, in order to ensure accurate results.

The results of the analysis are compared to the free-space behavior of the antenna in Figure 4, Figure 6 and Figure 7, showing very small differences between them. Regarding the antenna input impedance, a variation smaller than 2Ω is observed at the WPT port, while the effect on the data port is negligible. Similar conclusions can be extracted when analyzing the radiation pattern properties in both working modes.

### 2.5. Use of GenAI Tools

The authors did not use any GenAI tool for any purpose related to this work.

## 3. Results

### 3.1. Implementation

**Figure 9:**
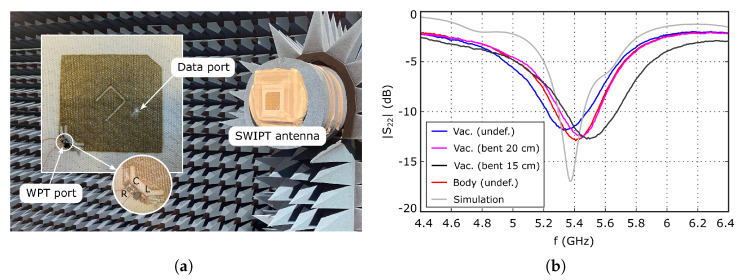
(**a**) Picture of the implemented prototype inside the anechoic chamber. (**b**) Measured input reflection coefficient $S_{22}$ at the data transfer coaxial port.

The multilayer fabric to fabricate the antenna, whose woven structure was schematized in Figure 2a, was woven using an industrial loom. Then, the antenna layout, including the radiating part and the T-match structure of the WPT port, was patterned on the top conductive layer through a laser cutting process. Although the precision of the laser cutting process can be considered to be ideal at the working frequencies when it is applied to conventional RF substrates, in this case, the geometrical accuracy is limited by the particular characteristics of the woven substrate, especially the diameter and the separation between conductive threads. A coaxial connector was attached to the antenna to test the performance of the data transfer mode. The inner and the outer conductors were connected to the top and bottom conductive layers, respectively, using conductive epoxy resin. Note that, in a final application, this connector is not strictly necessary, since the data signal source could be connected to the antenna using a conductive thread, increasing the degree of integration. A picture of the prototype is shown in Figure 9a.

To integrate the rectifier, the technique described in [19,20] was used. The rectifier was first implemented on a flexible polyimide strip metallized with silver-based conductive ink, and glued to a carrier thread. Next, the carrier thread was woven with the rest of the textile structure at the WPT port. In this way, the electrical contact between the rectifier and the WPT port terminals is made through the large number of contact points created when weaving the carrier thread. Furthermore, the diode and the other lumped components are not soldered to textile parts. Thus, the used technique provides the maximum degree of integration described to date for textile devices. Furthermore, when used in combination with the proposed indivisible multilayer textile structure, it allows us to improve the overall robustness. Hence, the proposed antenna is expected to be able to withstand the mechanical stress caused by the use under real-life conditions.

### 3.2. Measurement Setup

To experimentally characterize the performance of the proposed SWIPT antenna at the power transfer mode, a WPT transmitter composed of an RF signal source and a linearly polarized patch antenna, with gain $G_{TX}=4$ dB, was used. The RF source generates a signal with frequency $f_{RF}=2.4$ GHz and a power of 23 dBm, in order not to exceed the EIRP limits for indoor ISM transmitters. The distance between the transmitter antenna and the prototype was set to d=0.5 m, for which the free-space propagation loss at 2.4 GHz is about 34 dB. Thus, including an additional 3 dB loss factor due to the polarization mismatch between the linearly polarized transmitter antenna and the circularly polarized receiver antenna, the available power at the rectifier input is $P_{RF}\approx -10$ dBm. To estimate this power value, the 0 dB gain for the antenna under test obtained in simulation was considered. Note that the antenna gain at the WPT mode cannot be accurately measured because the input impedance at the WPT port is very different from 50Ω. At the receiver side, the rectified voltage $V_{DC}$ was directly measured at the resistor terminals, using a conventional multimeter, then the obtained DC power $P_{DC}$ was calculated.

Regarding the performance of the data link mode, the impedance matching at the coaxial port was evaluated using a vector network analyzer, while the radiation pattern characteristics, including the gain and the axial ratio, were measured in an anechoic chamber using standard measurement techniques.

### 3.3. WPT Mode Experimental Results

**Figure 10:**
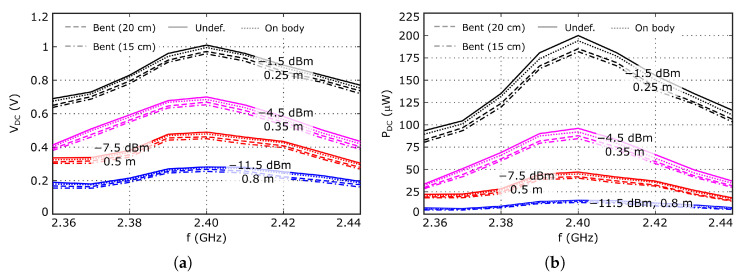
Measured performance of the antenna working at the WPT mode. (**a**) DC voltage. (**b**) DC power. The trace labels indicate the available power value at the antenna, which is about 3 dB larger than the available power at the rectifier input because of the polarization mismatch between the circularly polarized antenna under test and the linearly polarized transmitter antenna.

**Figure 11:**
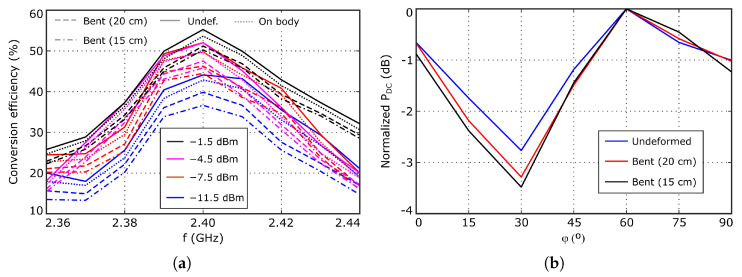
(**a**) Measured RF-DC conversion efficiency. (**b**) Evolution of the normalized measured DC power at the rectifier output for different rotation angles.

The experimental data presented in this section was measured in a non anechoic laboratory environment, in order to obtain results as close as possible to a real use scenario. The evolution of the DC voltage, $V_{DC}$, and power, $P_{DC}$, with the working frequency is shown in Figure 10a,b, respectively, while the computed RF-DC conversion efficiency is represented in Figure 11a. The nominal distance between the WPT transmitter and the antenna under test is d=0.5 m, for which the available RF power value at the rectifier input is $P_{RF}\approx -10$ dBm. At this distance, the maximum measured DC power value is $P_{DC}\approx 50\;\mu$W and the RF-DC conversion efficiency is slightly larger than 50%. The maximum value is reached at about 2.4 GHz, indicating optimum impedance matching between the antenna and the rectifier at that frequency. Furthermore, note that these traces are influenced by the frequency variation in the gain of both antennas, and the impedance mismatch effects.

The difference between the measured RF-DC conversion efficiency $\eta_{RF-DC}\approx 50\%$ and the 60% simulated value can be attributed to inaccuracies in the large-signal diode model used in simulation, or to potential deviations in the antenna gain and/or the input impedance, $Z_{WPT}$. Since the antenna gain at this working mode cannot be accurately evaluated, due to the input impedance value, $Z_{WPT}$, and the structure of the port, the indicated error sources cannot be evaluated separately. Note that, from the data represented in Figure 3, it is expected that a moderate variation in the antenna input impedance about the optimum point has relatively low impact on the overall conversion efficiency. Nevertheless, from a practical point of view, the most important magnitudes are the DC voltage and power at a given distance, as they determine the viability of the system, and these can be accurately measured. The antenna performance was also tested at 0.25, 0.35 and 0.8 m distances from the WPT transmitter, for which the available RF power at the antenna is about −1.5, −4.5, and −11.5 dBm, respectively. Note that the available power at the rectifier input is about 3 dB smaller than the previous values because of the polarization mismatch between the circularly polarized antenna under test and the linearly polarized transmitter antenna. The measured DC power at 0.8 m from the WPT transmitter is still higher than 15μW. The RF-DC conversion efficiency reduces with the available power at the rectifier input, because of the inherent characteristics of Schottky diodes.

To estimate the axial ratio, the antenna was rotated around the *z* axis from 0^*o*^ to 90^*o*^, with 15^*o*^ step, and the $P_{DC}$ value was evaluated for each rotation angle, considering $f_{RF}=2.4$ GHz. Since the incoming signal is linearly polarized, the power coupled to the antenna depends on the relative orientation between the antenna and the incoming electric field vector. In addition, the measured DC power at the rectifier output is proportional to the power coupled to the antenna. Thus, the evolution of the measured $P_{DC}$ value with the rotation angle allows us to estimate the antenna axial ratio as the difference between the maximum and the minimum values. The obtained results are presented in Figure 11b, showing a power variation smaller than 3 dB and, then, indicating that the WPT mode operates with circular polarization.

Finally, the antenna performance was evaluated under operating conditions closer to real wearable applications. First, it was bent along the x axis with two different radius, 20 and 15 cm, to analyze the impact of potential deformations. Furthermore, the antenna was placed over the torso of a volunteer to analyze the influence of human body proximity. The obtained results are represented in Figure 10 and Figure 11a, together with the data corresponding to the undeformed conditions, showing a stable behavior against moderate deformations, and that the antenna is very well isolated from the underlying objects, because of the naturally available ground plane.

### 3.4. Data Transfer Mode Experimental Results

First, the reflection coefficient at the coaxial port was measured using a vector network analyzer. The result is shown in Figure 9b, together with simulation data, showing relatively good agreement between them, especially when considering the particularities of the fully woven textile technology. Furthermore, it is observed that the measurement is stable against deformations, and that placing the antenna over the human body has negligible impact, as expected from the simulation data presented in Figure 4, Figure 6 and Figure 7.

**Figure 12:**
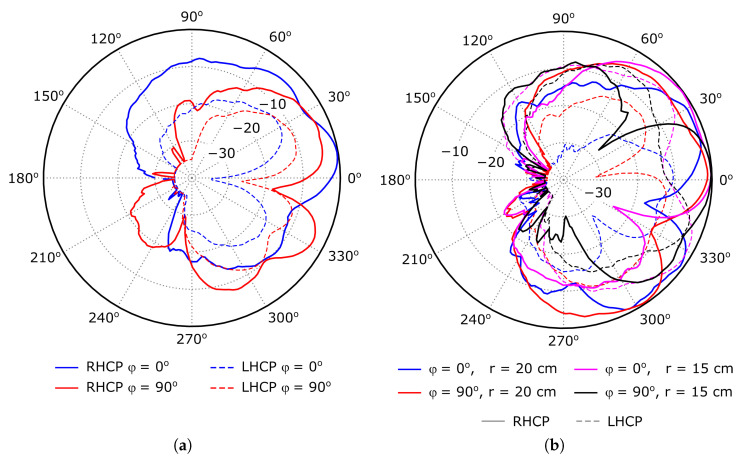
Normalized measured RHCP and LHCP components of the radiation pattern, evaluated at 5.4 GHz. For the sake of representation clarity, values smaller than −35 dB have been clipped to −35 dB. (**a**) Undeformed antenna. (**b**) Bent antenna, with radius r=15 and 20 cm.

The radiation pattern characteristics, including the gain and the axial ratio, were evaluated in an spherical-range anechoic chamber, using conventional antenna measurement techniques. The normalized measured RHCP and LHCP components of the radiation pattern, evaluated at 5.4 GHz, are represented in Figure 12. The antenna exhibits right-handed circular polarization in the data transfer mode, with a radiation pattern which is typical in modified patch antennas. The intercomparison technique was used to evaluate the gain, providing a −2.5 dB value, very close to that obtained in simulation. Furthermore, the impact of bending the antenna on the directional properties is negligible, but slightly increases the cross-polar levels.

**Figure 13:**
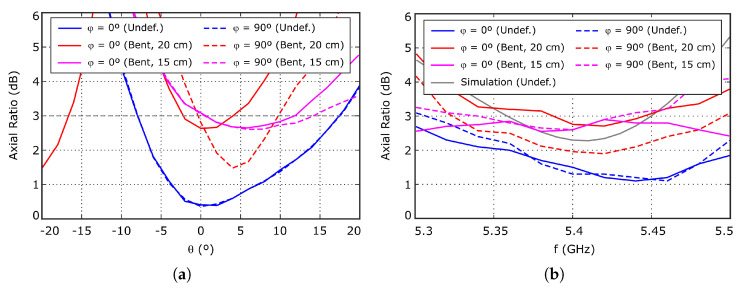
Measured axial ratio. (**a**) Evolution with θ, evaluated at 5.4 GHz. (**b**) Evolution with the working frequency, evaluated at $\theta =0^{o}$ direction.

Finally, the axial ratio was also experimentally characterized. Figure 13a represents the evolution of the axial ratio with the θ direction, evaluated at 5.4 GHz, for the two main cuts, φ=0 and 90^*o*^. On the other hand, the evolution of the axial ratio with the working frequency, evaluated at $\theta =0^{o}$ direction, is shown in Figure 13b. From the measurement data, the axial ratio slightly deteriorates when bending the antenna, which is coherent with the increased cross-polar levels shown in Figure 12b. Furthermore, note that the axial ratio of the undeformed antenna, evaluated at $\theta =0^{o}$ direction, does not depend on the φ cut, as expected. However, when considering the bent antenna, small differences are found between the measured values for φ=0 and 90^*o*^, due to the antenna deformation. Nevertheless, the value at 5.4 GHz is still about 3 dB in the worst case, providing acceptable polarization performance under moderate deformations.

## 4. Discussion

The measured performance of the presented fully woven textile SWIPT antenna was compared with the state of the art in SWIPT designs in Table 3. First, it should be considered that most of the recently reported antennas are implemented in conventional rigid RF substrates and include several metallization layers, which makes them unsuitable for wearable applications. On the other hand, refs. [12,26] describe textile nonwoven antennas, which are more adequate for wearable applications, but lack the advantages of fully woven technology described in this work.

Regarding the different SWIPT approaches, single-band and dual-band devices have been proposed. On the one hand, refs. [21,23,24,25,26] describe single-band antennas in which the power and the data transfer are performed in the same frequency band. In these cases, to isolate the two working modes, they operate with orthogonal linear or circular polarization. In the case of [23,25], both working modes can simultaneously operate with orthogonal linear polarization, but when using circular polarization, the device works as a rectenna, without data transfer capability. Furthermore, as also occurs with [28], additional circuitry is required to electronically switch between the different working modes. On the other hand, dual-band designs, in which the power and the data transfer modes operate at different frequency bands have also been described [12,28,29,30]. These designs can be seen as a pair of antennas, one optimized for either band, with some EM coupling between them, which simplifies the design process. However, their main drawback, with the exception of [12], is related to the use of complex structures with more than one metallization layer.

Most of the compared works report RF-DC conversion efficiency values larger than those this work. However, such efficiency values are achieved using very large available power at the rectifier input, in the 0–14 dBm range, which are unrealistic in practical scenarios, when considering the propagation losses and the EIRP limits at the transmitter. Furthermore, in the particular case of [12], in which an efficiency about 62%, with −10 dBm available power at the rectifier input is reported, it should be noted that the power transfer mode works in the 800 MHz band, in which the performance of Schottky diodes working as rectifiers is considerably better than in higher-frequency bands.

In this work, the first fully woven textile, dual-band, dual-mode, circularly polarized SWIPT antenna is presented. The power and the data transfer modes work at different frequency bands without requiring time multiplexing. Both of them are circularly polarized, in order to minimize the fading associated with polarization mismatch, caused by the potential variation in the relative orientation between the transmitter and the receiver antennas. This valuable characteristic is achieved using only one port for each mode, which allows their simultaneous operation, and avoids the use of additional switching circuitry. Regarding the RF-DC conversion efficiency, the measured value is larger than 50% under realistic operating conditions, when the available power at the rectifier input is −10 dBm. This value is comparable to the state of the art for woven and nonwoven textile devices, and even to antennas implemented in conventional substrates. Furthermore, all the indicated characteristics are achieved using a patch-like radiating structure whose layout requires only one metallization layer, apart from the ground plane layer, which simplifies the implementation in fully woven technology, and isolates its behavior from the underlying objects.

## 5. Conclusions

In this work, a textile fully woven, dual-band, dual-mode circularly polarized antenna for SWIPT applications in wearable devices is described. Although some devices with similar functionality were previously reported, they are not suitable for wearable applications because they were implemented in conventional rigid RF substrates. To the best of the authors’ knowledge, this is the first reported realization of a design combining all the indicated characteristics and implemented in fully woven textile technology, which contributes to advancing the state of the art for SWIPT antennas.

On the other hand, when compared to the only previously published design with similar functionality, but implemented in nonwoven technology [12], several key advantages are found in the present work. First, in [12], both working modes are linearly polarized, making its performance highly dependent on the relative orientation with respect to the transmitter. Secondly, the radiating structure used for the power transfer mode is similar to a dipole, whose radiation properties are very sensitive to the underlying objects. Furthermore, it was implemented in nonwoven textile technology, which lacks some of the advantages of the fully woven alternative.

Regarding the achieved electrical performance, it is comparable to the state of the art for textile devices and, even, to those implemented in conventional rigid RF substrates, while preserving the advantages of fully woven technology, i.e., flexibility, robustness, ease of integration with larger fabric pieces, and the possibility of large-scale low-cost production using processes from the textile industry. In this way, the fully woven textile technology represents a promising approach to address the challenges existing in the design of wearable devices. Furthermore, the presented results can be applied to other fields which share some common issues, such as the development of massive sensor networks or IoT applications. 

## Figures and Tables

**Table 1:** Optimized geometrical parameters, in mm.

**Parameter**	$L_{p}$	$C_{1}$	$C_{2}$	*g*	$l_{1}$	$l_{2}$	$l_{u1}$
**Value**	42	4.9	5.6	1.5	6	4.5	11.3
**Parameter**	$l_{u2}$	$w_{u}$	$d_{x}$	$d_{y}$	$h_{d}$	$h_{c}$	
**Value**	8.4	9.6	9.8	3.7	0.81	0.001	

**Table 2:** Electrical parameters of the different layers in the human body model.

	Skin	Fat	Muscle	Bone
$\varepsilon_{r}$	37	5.2	51.5	10.8
σ (S/m)	2.02	0.16	2.56	0.61
Thickness (mm)	3	7	20	10

**Table 3:** Comparison with the state of the art.

Ref.	WPT/Data freq. (GHz)	Polarization	Power (dBm)	Efficiency (%)	Working Modes	Technology
[23]	2.4	Dual Linear/Circular	0	45	SWIPT/Rectenna	Rigid PCB
[24]	5.8/6.1	Dual Linear	14	65	SWIPT	Rigid PCB
[25]	5.7/5.8	Dual Linear/Circular	0.5	51	SWIPT/Rectenna	Rigid PCB
[28]	4.2-6.7	Dual Linear/Dual Circular	10	71	SWIPT/Rectenna	Rigid PCB
[29]	5.8/2.4	Dual Circular	12	63	SWIPT	Rigid PCB
[30]	5/5.7	Dual Linear	5	51	SWIPT	Rigid PCB
[26]	2.4	Dual Linear	2	74	SWIPT	Nonwoven textile
[12]	0.83/2.4	Dual Linear	−10	62	SWIPT	Nonwoven textile
[21]	2.4	Dual Circular	−10	50	SWIPT	Fully woven textile
**This work**	2.4/5.4	Dual Circular	−10	50	SWIPT	Fully woven textile

## Data Availability

The original data presented in the study is openly available with the access links https://zenodo.org/records/17977124 and https://hdl.handle.net/10651/81677, both accessed on 16 December 2025.

## Abbreviations

The following abbreviations are used in this manuscript:

DC	Direct Current
EM	Electromagnetic
HB	Harmonic Balance
LF	Low Frequency
LHCP	Left-Handed Circular Polarization
RF	Radio Frequency
RFID	Radio Frequency Identification Technique
RHCP	Right-Handed Circular Polarization
SWIPT	Simultaneous Wireless Information and Power Transfer
UHF	Ultra High Frequency
WPT	Wireless Power Transfer
